# Computational understanding and experimental characterization of twice-as-smart quadruplex ligands as chemical sensors of bacterial nucleotide second messengers

**DOI:** 10.1038/srep33888

**Published:** 2016-09-26

**Authors:** Jie Zhou, Benjamin T. Roembke, Gabor Paragi, Aurélien Laguerre, Herman O. Sintim, Célia Fonseca Guerra, David Monchaud

**Affiliations:** 1 Department of Chemistry and Center for Drug Discovery, Purdue University, West Lafayette, IN 47907, USA; 2 Graduate Program in Chemistry, University of Maryland, College Park, MD 20742, USA; 3 Department of Theoretical Chemistry, Vrije Universiteit Amsterdam, The Netherlands; 4 MTA-SZTE Supramolecular and Nanostructured Materials Research Group, Szeged, Hungary; 5 Institut de Chimie Moléculaire, ICMUB CNRS UMR6302, UBFC Dijon, France

## Abstract

A twice-as-smart ligand is a small molecule that experiences a structural switch upon interaction with its target (*i.e.*, smart ligand) that concomitantly triggers its fluorescence (*i.e.*, smart probe). Prototypes of twice-as-smart ligands were recently developed to track and label G-quadruplexes: these higher-order nucleic acid structures originate in the assembly of four guanine(G)-rich DNA or RNA strands, whose stability is imparted by the formation and the self-assembly of G-quartets. The first prototypes of twice-as-smart quadruplex ligands were designed to exploit the self-association of quartets, being themselves synthetic G-quartets. While their quadruplex recognition capability has been thoroughly documented, some doubts remain about the precise photophysical mechanism that underlies their peculiar spectroscopic properties. Here, we uncovered this mechanism *via* complete theoretical calculations. Collected information was then used to develop a novel application of twice-as-smart ligands, as efficient chemical sensors of bacterial signaling pathways *via* the fluorescent detection of naturally occurring extracellular quadruplexes formed by cyclic dimeric guanosine monophosphate (c-di-GMP).

The interest for the discovery of ever more efficient molecular probes to detect G-quadruplexes in a convenient manner is mounting^1^, compounded by accumulating evidences of quadruplex roles in many therapeutic areas including cancers^2^,^3^,^4^ and neurodegenerative^5^,^6^, viral or infectious diseases^7^. One of the conspicuous features of quadruplex architectures is their high polymorphism^8^,^9^, which is counterbalanced by the structural uniqueness of their ligand binding site, primarily the accessible external G-quartet^10^,^11^. Therefore, a way to design multitarget quadruplex ligands relies on the fine-tuning of broad aromatic molecules, displaying the somewhat paradoxical properties of being water-soluble (usually cationic, for interacting with negatively charged native nucleic acids) and hydrophobic (for enhancing interactions with the accessible G-quartets)^12^,^13^. This apparent duality explains why really efficient quadruplex ligands (*i.e.*, water soluble and stable, displaying high affinity and selectivity for quadruplexes, etc.) are still sparse in the literature, despite hundreds of new candidates reported each year. Also, it explains why the design of effective quadruplex-selective fluorescent probes is challenging, since already demanding candidates must be additionally endowed with ideally suited photophysical properties (turn-on fluorescence, photostability, etc.), which add an extra level of complexity.

We recently addressed some of these issues using biomimetic quadruplex ligands^14^,^15^, the structure of which comprises four synthetic guanines around a central template, which can assemble to form an intramolecular G-quartet. These compounds termed TASQ (for template-assembled synthetic G-quartet)^16^ are conformationally flexible (the four guanines can be assembled or not) and their quadruplex affinic conformation is triggered by the interaction with targets only: the like-likes-like recognition that takes place between native (quadruplex) and synthetic (ligand) G-quartets makes TASQ pioneering smart quadruplex ligands^17^,^18^. When the template is fluorogenic (a naphthalene or a pyrene)^19^,^20^, TASQ behave as a sensor whose fluorescence is also triggered by the interaction with their targets only: these new generation TASQ are thus concomitantly smart quadruplex ligands and smart fluorescent probes, that is, the unique prototypes of twice-as-smart quadruplex ligands.

The very first example of a twice-as-smart quadruplex ligand was PyroTASQ (Fig. 1)^19^, in which guanines are assembled around a tetrasubstituted pyrene core. The interactions between PyroTASQ and quadruplexes were thoroughly investigated *in vitro*, not only in terms of affinity (for both DNA and RNA quadruplexes) and selectivity (over duplex-DNA), but also for its ability to fluorescently label quadruplexes with efficacy (high S/N ratio, up to 90-fold) and selectivity (over duplexes, again). However, the mechanism that underlies the quite peculiar photophysical properties of PyroTASQ remained to be fully understood: we address here this question *via* complete molecular modeling investigations and exploit the resulting findings to develop a new PyroTASQ application as chemical sensor of bacterial signaling pathways.

## Results

### PyroTASQ efficiently labels DNA and RNA quadruplexes

The ability of PyroTASQ to interact with nucleic acids was firstly studied at two levels, *i*- its affinity for both DNA- and RNA-quadruplexes and its selectivity over duplexes, and *ii*- its capability of fluorescently label quadruplexes and its selectivity over duplexes. The results collected through FRET-melting^21^,^22^, an *in vitro* assay routinely used for quantifying the ligand/DNA interactions in a convenient manner, indicate that PyroTASQ displays a high affinity for a series of quadruplex-forming oligonucleotides (QFO) including DNA sequences found in the human telomeres^23^ and in the promoter region of myc and kit genes^3^ (F21T, F-myc-T and F-kit-T, respectively), and RNA sequences found in telomeric transcripts and in the untranslated region of mRNA of the telomeric repeat binding factor 2 and vascular endothelial growth factor genes (L-TERRA, L-TRF2 and L-VEGF, respectively)^24^,^25^. Conversely, PyroTASQ does not interact with duplexes, thus demonstrating a high quadruplex-selectivity. These results were supported by a series of *in vitro* fluorescent titrations carried out with unlabeled QFO (myc, kit, SRC, TERRA) and duplexes (ds17, ds26 and calf-thymus DNA), which were furthermore confirmed by polyacrylamide gel electrophoresis (PAGE) analyses. Collectively, these results showed that PyroTASQ is an efficient and quadruplex-selective fluorescent probe.

**Figure 1:**
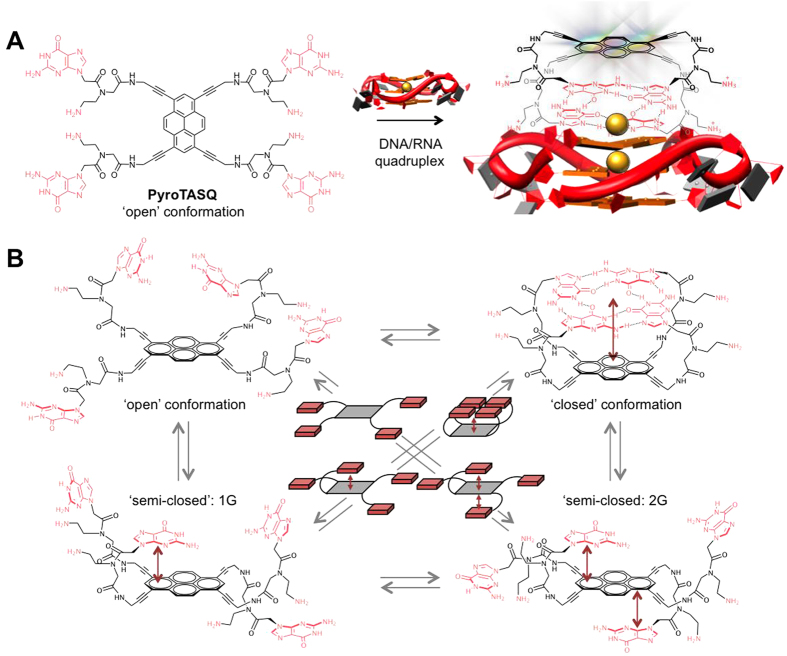
Conformational pluralism of PyroTASQ. (**A**). Conformational switch promoted by its interaction with quadruplexes: the formation of the intramolecular G-quartet triggers the fluorescence of the pyrene template. (**B**) Schematic representation of the possible ‘open’, ‘semi-closed’ (with one or two guanines stacking on the template, 1G and 2G, respectively) and ‘closed’ conformations of PyroTASQ.

The origins of the photophysical properties of PyroTASQ rely on its conformational pluralism: alone in solution, PyroTASQ adopts its ‘open’ conformation in which guanines are unassembled, and the interaction with quadruplexes triggers the folding of PyroTASQ in its ‘closed’ conformation, in which the intramolecular G-quartet is formed (Fig. 1). It was proposed that the PyroTASQ structural switch restores the fluorescence of its pyrene template as a result of a redistribution of the guanines’ electrons (from isolated guanines to guanines embedded in a G-quartet) that relieves the template from its electronic restraint (an intramolecular photoinduced electron transfer, iPET). We demonstrate herein *via* deep computational analyses that this mechanism is in fact more complex than initially anticipated.

### Conformational analysis of PyroTASQ

**Figure 2:**
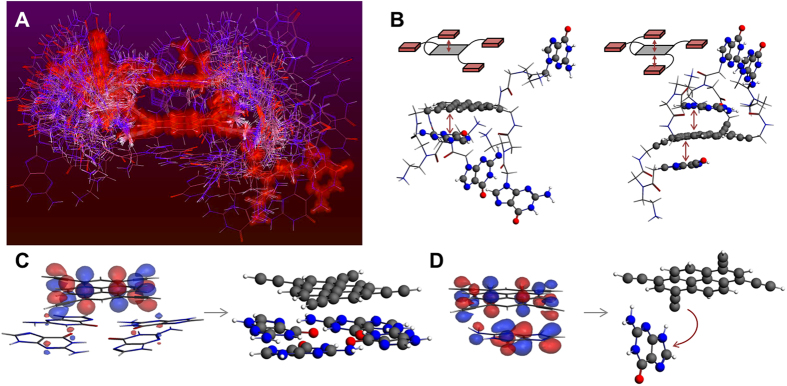
Computational analyses of the conformations of PyroTASQ. (**A**) Overlay of the 40 most stable PyroTASQ conformers after geometrical optimizations of PyroTASQ at molecular mechanical level; the most stable conformer is highlighted in red. (**B**) Two ‘semi-closed’ conformations 1G and 2G optimized *via* DFT analysis at BLYP-D3/DZP level. (**C**,**D**) Ground (left) and excited (right) states of a simplified ‘closed’ (**C**) and ‘open’ (**D**) conformations comprising only pyrene and G-quartet (**C**) or guanine (**D**) moieties optimized at BLYP-D3/TZP level of DFT gas phase calculation. Excitation was calculated with the Davidson method.

The elucidation of the quenching mechanism of the PyroTASQ fluorescence firstly requires a comprehensive analysis of the conformations it may adopt in solution. To this end, geometrical optimizations were undertaken at molecular mechanical (MM) level and the energetically most preferred 40 structures (Fig. 2A) were selected to represent the conformational space, among which none adopts the ‘closed’ conformation. Quite interestingly, the conformer with the lowest MM level energy, highlighted in red in Fig. 2A, is characterized by a guanine lying atop the pyrene, thus highlighting a peculiar relationship between the guanines and the template. To further investigate this, three conformations were optimized *via* density functional theory (DFT) analyses performed at the BLYP-D3/DZP level, including a conformation in which one guanine stacks onto the pyrene, another one in which two guanines stack on both sides of the pyrene, and the last one that corresponds to the ‘closed’ conformation (guanine quartet stacked on pyrene, see Fig. 1B). Both gas phase and implicit solvent COSMO calculations show that the two ‘semi-closed’ conformations are the most stable ones: the relative energies of the optimized structures with one, two or four guanines stacking on the pyrene template (*i.e.*, 0.2, 0.0 and 1.6 kcal.mol^−1^ in the gas phase, 13.2, 0.0 and 13.5 kcal.mol^−1^ in implicit water at the BLYP-D3/TZ2P//BLYP-D3/DZP level of theory) indicate that the most stable conformer comprises two stacked guanines (Fig. 2B). It is noteworthy that implicit solvent has a large impact on the determined energies, but it does not change significantly the geometry of the optimized conformations. Altogether, these results are in line with the experimental NMR evidences, which demonstrate that PyroTASQ primarily adopts its ‘open’. (or ‘semi-closed’, as opposed to ‘closed’) conformation when free in solution.

Subsequently, simplified PyroTASQ models comprising solely guanine and pyrene moieties (that is, devoid of aliphatic arms) were further optimized *via* DFT analyses at the BLYP-D3/TZ2P level with Davidson excitation calculation method. Several conformers were studied in both ground and excited states: three of them were modelling the ‘closed’ conformations (*i.e.*, with one or two G-quartets stacking on one side of the pyrene, to mimic PyroTASQ alone or in interaction with an accessible G-quartet of a quadruplex, respectively, without or with physiologically relevant K^+^ cation, one example is seen in Fig. 2C), and two models were mimicking the ‘semi-closed’ conformation (*i.e.*, with one or two guanines stacking on both sides of the pyrene, one example is seen in Fig. 2D). First, ground state optimizations were performed to decipher the HOMO and LUMO distribution of all conformers (*vide infra*); in each instance, the pyrene template and both isolated guanines and G-quartets were found coplanar, on the basis of strong π-stacking interactions. Second, the excited state geometries of two selected models (mimicking both the ‘semi-closed’ and ‘closed’ conformations, with one guanine and one G-quartet stacking onto the pyrene, respectively) were determined: the two conformers behave differently since the isolated guanine progressively flips out of the pyrene plane to end in a perpendicular position in gas phase, while the G-quartet remains parallel to the template (Fig. 2D). This isolated guanine motion is of utmost importance for elucidating the quenching mechanism of PyroTASQ (*vide infra*) since it opens the possibility of mechanical thermal relaxation upon photophysical excitation.

### Orbital analysis of PyroTASQ

**Figure 3:**
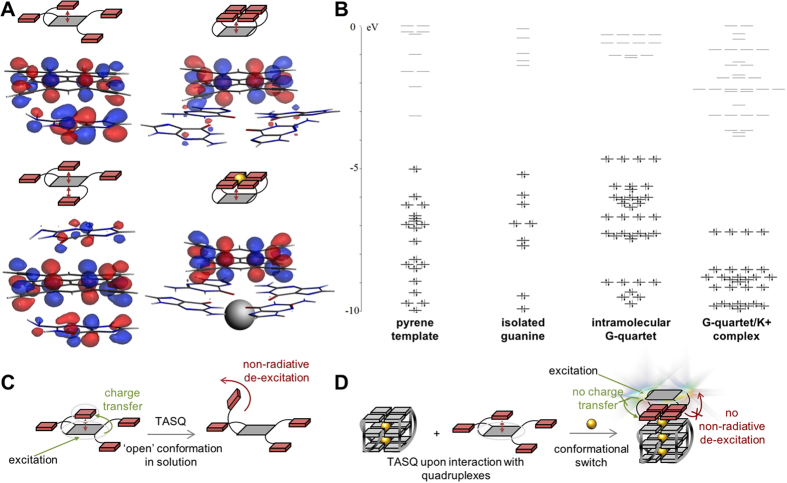
Molecular orbital analyses of the conformations of PyroTASQ. (**A**) Determination of the HOMO of two simplified ‘open’ models (left) and two simplified ‘closed’ models (right). (**B**) Molecular orbital levels of pyrene, guanine, G-quartet and G-quartet/K^+^ complex. (**C**,**D**) Schematic representation of the newly postulated mechanism that explains the turn-on fluorescence properties, that is, the smart nature of PyroTASQ.

The mechanism underlying the photophysical behavior of PyroTASQ was thus investigated on the basis of the relative energies of highest occupied molecular orbitals (HOMO) of four of the five aforementioned simplified models. As seen in Fig. 3A, the striking difference between the ‘semi-closed’ and ‘closed’ conformation models is the distribution of the HOMO that are located on both fragments for the ‘semi-closed’ models while located almost exclusively on the pyrene for the ‘closed’ models. These calculations thus indicate that while the HOMO of the ‘semi-closed’ models originate in a combination of the HOMO from both isolated guanine and pyrene fragments, that of the ‘closed’ models rest primarily on the HOMO of the pyrene alone. These findings are critical for deciphering the photophysical properties of PyroTASQ since the charge transfer that occurs between the guanine and pyrene moieties during spectroscopic investigations is most probable in the ‘semi-closed’ models. In other words, the folding of PyroTASQ in its ‘closed’ conformation, that is, when it interacts with quadruplexes, breaks the electronic relationship between its two constitutive aromatic units and makes the pyrene behaving (*i.e.*, fluorescing) as alone in solution. This could thus explain why PyroTASQ is a turn-on probe, which fluoresces only in the presence of quadruplexes.

The initially formulated theory related to the quenching mechanism of PyroTASQ was based on a redistribution of the guanines’ electrons when guanines self-assemble in a G-quartet that relieves the pyrene from an iPET electronic restraint (*vide supra*). This theory therefore requires: *i*- an energy level of the HOMO of the isolated guanine in between the HOMO and LUMO of the pyrene template, to allow for the excited electron (from pyrene) to go back to its initial HOMO through that of the guanine in a non-radiative manner; *ii*- a drop of the HOMO when guanines are embedded in a G-quartet, to preclude electron exchange between pyrene and guanine moieties. To investigate this, the energy level diagrams of the pyrene, an isolated guanine, a cation-less G-quartet and a G-quartet/K^+^ complex were established (Fig. 3B). These calculations clearly show that the initially postulated mechanism cannot occur here: indeed, the HOMO of isolated guanines are not found in between the HOMO and LUMO of the pyrene and are even slightly more negative than that of the template. Also, the formation of G-quartet provides HOMO even higher in energy than both pyrene and isolated guanine. However, these calculations show that, as initially thought, the K^+^-promoted formation of the G-quartet, which is certainly the biologically relevant conformer, lowers significantly the HOMO of embedded guanines. Altogether, these data indicate that the quenching mechanism of PyroTASQ is in need of a deep rethink.

### Quenching mechanism of PyroTASQ

In light of the aforementioned calculations, we thus revisit the mechanistic basis of the photophysical properties of PyroTASQ. On one hand, ground state optimizations of the ‘semi-closed’ and ‘closed’ models show that the corresponding HOMO are differently located, on the whole structure for the ‘semi-closed’ models and mostly on the pyrene for the ‘closed’ models. On the other hand, ground and excited state optimizations show that, while the ‘closed’ conformation models do not structurally change upon excitation (*i.e*., the quartet and pyrene remain coplanar), the ‘semi-closed’ model experiences deep structural reorganizations, with the isolated guanine that tries to move from a planar to a perpendicular position with respect to the template plane. Collectively, these data thus allow for proposing a new mechanism (Fig. 3C,D): alone in solution, PyroTASQ adopts an ‘semi-closed’ conformation in which at least one but probably two guanines stack on the pyrene template. Upon excitation, the HOMO of both pyrene and guanine moieties are involved; however, isolated guanines can flip out of the pyrene plane, thus offering a vibrational motion that can dissipate energy in a non-radiative manner. Additionally, charge transfer can occur between pyrene and guanines, thus offering a way to indirectly dissipate the energy accumulated by the pyrene through guanine motions. This explains the quenching mechanism. Conversely, upon interaction with quadruplexes, PyroTASQ folds into its ‘closed’ conformation, in which the intramolecular G-quartet is lying atop the pyrene template. Owing to the fact that *i*- the corresponding HOMO is almost exclusively located on the pyrene, *ii*- this HOMO distribution precludes charge transfer between guanine and pyrene moieties, and *iii*- guanines are firmly entrapped in the G-quartet (that is, they cannot easily escape from the quartet due to the chelation of K^+^ cation)^26^, the energy accumulated by the pyrene is primarily dissipated through fluorescence emission. This explains the mechanism that prevents quenching. Collectively, computational analyses offer a brand new appreciation of the photophysical properties of twice-as-smart quadruplex ligands.

### PyroTASQ is an *in vitro* detection tool

**Figure 4:**
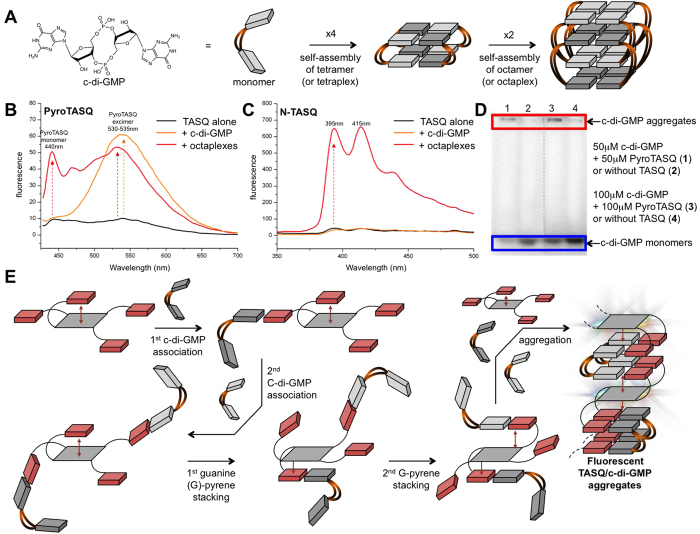
(**A**) Chemical structure of c-di-GMP and schematic representation of its higher-order folding into tetraplex and ocatplex. (**B**,**C**) Fluorescence titration results of experiments carried out with 10 μM TASQ (PyroTASQ, **B** and N-TASQ, **C**) alone in solution (black lines) or upon addition of monomeric c-di-GMP (10 μM, orange lines) or octaplexes (2.5 μM, red lines) in Tris-HCl buffer (pH 7.5). (**D**) Gel shift assay of ^32^P-c-di-GMP (50 μM lanes 1 and 2, 100 μM lanes 3 and 4), in absence (lanes 1 and 3) or presence of PyroTASQ (50 μM lane 2, 100 μM lane 4), for experiments carried out at 140 V during 20 min in 8% polyacrylamide gel, in 90 mM TBE buffer (pH 7.5) supplemented with 100 mM KCl. (**E**) Schematic representation of the PyroTASQ/monomeric c-di-GMP association that results in fluorescent aggregates.

In light of its quite unique turn-on fluorescence properties, PyroTASQ is used for the detection of quadruplexes *in vitro via* both fluorescence titrations and PAGE analyses. However, the propensity of PyroTASQ to self-assemble through pyrene-pyrene association makes its use for tracking biologically relevant DNA and RNA quadruplexes in living cells challenging (higher concentrations are required), as it aggregates in extracellular spaces and on cell membranes. For this reason, a cell-compatible twice-as-smart quadruplex ligand was developed, NaphthoTASQ (or N-TASQ), owing to the reduced propensity of naphthalene templates to self-associate. We believe anyway that the spectroscopic properties of PyroTASQ deserve to be further exploited, especially now we have a more precise idea of the conformation it may adopt in solution. We thus turned our attention to naturally occurring extracellular quadruplexes formed by cyclic dimeric guanosine monophosphate (c-di-GMP, Fig. 4).

The c-di-GMP is a ubiquitous bacterial second messenger that controls various key processes including biofilm formation, motility and sporulation^27^,^28^,^29^. The importance of c-di-GMP in bacterial life has lead to development of research programs aiming at better understanding the intricacies of the dinucleotide signaling pathways. Central to the advancement of these investigations was the development of sensors used to detect c-di-GMP, study their interactions with putative partners and determine the activity of related enzymes (c-di-GMP synthases or phosphodiesterases). We recently discovered that simple intercalators such as thiazole orange (TO) or proflavine could act as molecular chaperones and promote the aggregation of c-di-GMP into biologically inert G-quartet–based polymorphs^30^,^31^,^32^,^33^. Conveniently, the fluorescence of TO is enhanced upon binding that provides a straightforward mean to detect the messenger. However, simple intercalators like TO suffer from restrictions, primarily their indiscriminate affinity for nucleic acids whatever their secondary structures (single strand, duplex, quadruplex), which preclude further technological developments in real biological conditions. We thus decided here to assess whether twice-as-smart quadruplex ligands might be valuable sensors to interact with, aggregate and label efficiently c-di-GMP.

To this end, the interactions of both PyroTASQ and N-TASQ with either monomeric or aggregated c-di-GMP were studied *via in vitro* fluorescence titrations. Monomeric c-di-GMP can indeed fold into higher-order structures when diluted in water at millimolar concentrations, notably quadruplexes comprising eight intertwined c-di-GMP consequently termed octameric c-di-GMP complexes, or octaplexes (Fig. 4A)^34^,^35^,^36^,^37^,^38^. The structures are stable enough to be subsequently diluted at micromolar concentrations without disassembly. Fluorescence titrations were performed with both TASQ (10 μM) and either monomeric c-di-GMP (20 μM) or octameric c-di-GMP (2.5 μM) in Tris-HCl buffer (pH 7.5). Results seen in Fig. 4B,C highlight the different behavior of both TASQ: while the fluorescence of N-TASQ is turned-on by octaplexes only, that of PyroTASQ is enhanced upon interaction with both monomers and octaplexes. Also, the fluorescence of PyroTASQ centered on 530 nm, which is characteristic of the formation of pyrene-pyrene excimers. Collectively, these results suggest that, while N-TASQ binds solely to pre-folded c-di-GMP–based quartets, PyroTASQ might provoke c-di-GMP aggregation in higher-order c-di-GMP/TASQ aggregates, making it more promising than N-TASQ in this therapeutic area. A hypothesis that might explain why PyroTASQ strongly interacts with monomers can be found in the aforementioned molecular modeling study: when in its ‘open’ conformation, the ligand might use its two or three guanines that are not stacked on the pyrene template to recruit c-di-GMP *via* guanine-guanine associations (Fig. 4E). To further investigate this, PAGE experiments were performed with radioactive ^32^P-c-di-GMP: results seen in Fig. 4D clearly demonstrate the ability of PyroTASQ to promote the aggregation of c-di-GMP since it barely migrates out of the wells in presence of PyroTASQ (at 1:1 c-di-GMP/TASQ ratio, at either 50 or 100 μM concentration), while it undergoes a fast-paced migration when alone in the wells.

**Figure 5:**
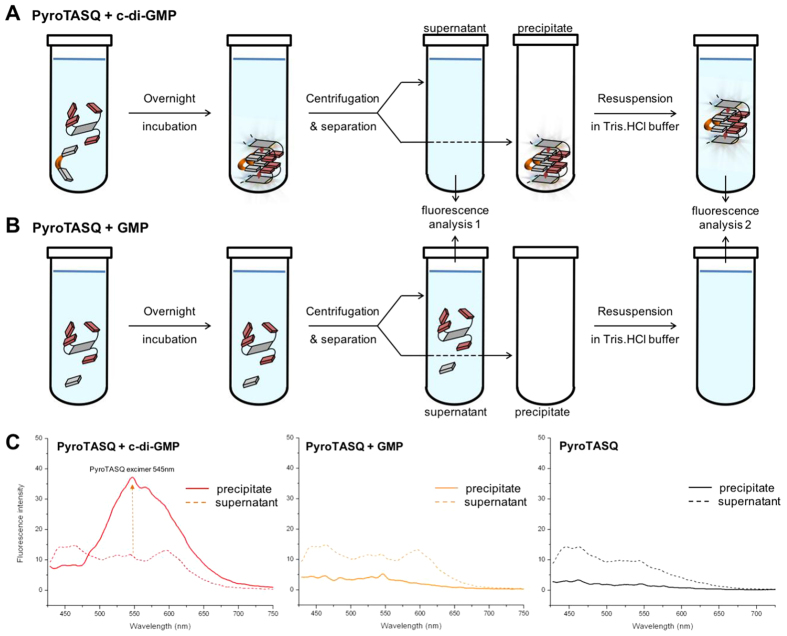
(**A**,**B**) Schematic representation of the multistep experimental setup for (**A**) isolating and visualizing the PyroTASQ/c-di-GMP aggregates and (**B**) a control experiment carried out with PyroTASQ and GMP. This protocol comprises overnight incubation at 4 °C of TASQ/GMP mixtures, followed by a centrifugation step (15 min, 14,000 rpm at 4 °C) and phase separation: the supernatant is carefully removed and its fluorescence is measured (*i.e.*, fluorescence analysis 1), and the precipitate is taken up in Tris-HCl buffer, manually harvested, and the fluorescence of the resulting solution is measured (*i.e.*, fluorescence analysis 2). (**C**) Fluorescence results of experiments carried out with 50 μM PyroTASQ and c-di-GMP (50 μM, red lines), GMP (50 μM, orange lines) or alone in solution (black lines) or in Tris-HCl buffer (pH 7.5): dashed lines correspond to the fluorescence of the supernatant (*i.e.*, fluorescence analysis 1) while plain lines correspond to the fluorescence of the resuspended precipitate (*i.e.*, fluorescence analysis 2).

To lend further credence to this new application of twice-as-smart ligand (*i.e.*, promoting the c-di-GMP aggregation), we decided to investigate whether PyroTASQ might both trigger ci-d-GMP precipitation and fluorescently label the resulting nanoassemblies. To this end, a multistep experimental protocol was set up (schematically represented in Fig. 5A,B) that allows for *i*- the formation of c-di-GMP/TASQ aggregates by overnight incubation, followed by *ii*- a physical separation by centrifugation of unaggregated and TASQ-promoted aggregated c-di-GMP, and *iii*- a systematic fluorescence analysis of the resulting fractions. Experiments were performed with c-di-GMP (50 μM) and PyroTASQ (50 μM) (Fig. 5A), along with GMP (50 μM) and PyroTASQ (50 μM) (Fig. 5B) as well as with PyroTASQ alone (50 μM) as control experiments. As seen in Fig. 5C, while residual fluorescence of PyroTASQ is seen in the three supernatant solutions, the sole fluorescence response in the precipitate fractions is obtained for the c-di-GMP/PyroTASQ mixture, indicating that only these two partners provide easily detectable fluorescent aggregates in the micromolar range. Altogether, these results highlight that PyroTASQ is a promising sensor, capable of disrupting dinucleotide signaling pathways (by promoting the aggregation of the c-di-GMP messenger) and offering a convenient way to detect therapeutically useful biomarkers (by fluorescently labeling the resulting c-di-GMP/TASQ aggregates).

## Discussion.

The quest for ever more versatile small molecule fluorophores is currently the focus of much attention since modern therapeutic approaches, whatever the therapeutic area, rely on mechanistic insights into drug activity at cellular level by fluorescence techniques^39^. Given that DNA and RNA quadruplexes are now considered as promising genetic disease markers (cancers, neurological disorders)^2^,^3^,^4^,^5^,^6^, massive efforts have been recently invested to design fluorescent dyes to label human cell-relevant quadruplexes. However, the more we discover the rich quadruplex-related biology, the more we look for and find quadruplex-forming sequences all along the phylogenetic tree of the living organisms. For instance, bacterial and viral quadruplexes are now considered as valuable targets for therapeutic interventions aiming at harnessing pathogenic virulence^7^, as demonstrated by recent and elegant works from research teams of J.S. Hartig^40^,^41^,^42^ and S. Richter^43^,^44^,^45^,^46^ for instance.

Here, we keep on expanding the repertoire of chemical tools to study bacterial quadruplexes through quadruplex-like structures comprising eight intertwined c-di-GMP termed octaplexes, which are central to bacterial signaling pathways. We show that quadruplex-selective fluorophores of the TASQ family –chiefly PyroTASQ– are useful not only for tracking but also for promoting the aggregation of c-di-GMP. TASQ thus offer a unique theranostic opportunity to fight against bacterial invasion, concomitantly disrupting the signaling networks by hijacking the ubiquitous bacterial second messenger c-di-GMP in silent aggregates (that is, impending biofilm formation) and providing easily readable fluorescent outputs as a results of the aggregate formation (that is, localizing where the bacterial invasion occurs). Admittedly, the theoretical and experimental *in vitro* results presented here represent a first insight only into the antipathogenic theranostic possibilities of twice-as-smart ligands. They are nonetheless important since they provide a better understanding of the structural and electronic properties of TASQ in solution. Our results provide also a bright illustration of the subtle differences in behavior between two closely related TASQ: N-TASQ labels pre-folded octaplexes only while PyroTASQ promote c-di-GMP aggregation and leads to fluorescent c-di-GMP/TASQ aggregates. These observations remain to be fully understood and we need to disentangle geometrical and electronic considerations, along with the way they govern the interactions between TASQ and c-di-GMP, octaplexes or both: *e.g.*, the two templates (naphthalene *versus* pyrene) organize the intramolecular G-quartet differently (2 sets of *ortho*-positioned arms on a 10-membered ring for N-TASQ *versus* 2 sets of *meta*-positioned arms on a 14-membered ring for PyroTASQ, respectively), that is, with different steric constraints and hence stability; also, the two templates do not allow for the same π-stacking interactions with isolated guanines or folded quartets; etc. The contributions of these steric and electronic parameters have now to be deciphered *via* further experimental and molecular modeling approaches; our results provide a solid basis for these investigations and beyond this, highlight the unique properties of PyroTASQ and its promising potential as theranostic agent. Results presented herein thus lay solid foundations for future studies that could help understand how small molecules could be useful for the concomitant induction and detection of quadruplex-based bacterial c-di-GMP aggregates. Beyond c-di-GMP sensing, the aggregation of this dinucleotide using a smart G-quadruplex ligand is interesting and could have nanotechnological or biomedical application. Higher order aggregates of c-di-GMP and aromatic intercalators have been shown to be resistant to phosphodiesterase hydrolysis, implying that one could perturb c-di-GMP signaling with small molecules^31^,^32^. Prior report, however, utilized thiazole orange or proflavine in aggregating c-di-GMP but these molecules are non-specific and bind to various nucleic acids. Smart ligands could aggregate c-di-GMP to potentially perturb c-di-GMP signaling without affecting other nucleic acids and therefore represent more promising scaffolds to develop c-di-GMP “aggregators”.

## Experimental part

### Materials

C-di-GMP was synthesized using previously described techniques and purified using reverse phase HPLC^47^. All other reagents were obtained from commercial sources and used without further purification. C-di-GMP concentration was determined via UV absorbance measurement at 253 nm, with extinction coefficient: 28,600 M^−1^cm^−1^. All fluorescence measurements were made on a Varian Cary Eclipse spectrophotometer, with λ_ex_ 410 nm (slit 5 nm) and λ_em_ 420–750 nm for PyroTASQ and λ_ex_ 276 nm (slit 5 nm) and λ_em_ 286–500 nm for N-TASQ.

### Sample preparation for c-di-GMP fluorescence titration

All samples were prepared as follows: 50 mM Tris-HCl (pH 7.5), 100 mM KCl and c-di-GMP stock solutions were combined and mixed thoroughly. The solutions were heated (using a heating block) to 95 °C and kept at 95 °C for 5 min. The samples were then allowed to cool down at room temperature for 15 minutes before smart ligands (PyroTASQ or N-TASQ, 10 μM) were added to a desired final concentration. Samples were then incubated at 4 °C for 12 h before fluorescence measurements were taken.

### Sample preparation for octaplex fluorescence titration

Following literature protocol^37^, monomeric c-di-GMP (2 mM) was converted into the octaplex form in a buffer containing 250 mM KCl, 5 mM sodium phosphate and 10 mM EDTA (pH = 7.5) and kept at 4 °C for 24 h. The concentration of octameric c-di GMP was estimated to be 250 μM as 8 molecules of c-di-GMP form octaplex. Solutions of c-di-GMP octaplex, 50 mM Tris-HCl (pH 7.5), 100 mM KCl and smart ligands (PyroTASQ or N-TASQ, 10 μM) were then combined and mixed well before fluorescence measurement.

### Gel shift assay

Radiolabeled ^32^P-c-di-GMP was prepared as previously described, using the mutated DGC WspRD70E and ^32^P-GTP^48^. Radiolabeled c-di-GMP was prepared as a mixture of “hot (^32^P)” and “cold (^31^P)” c-di-GMP (stock concentration of 500 μM). Radiolabeled c-di-GMP and 50 mM Tris-HCl buffer solution (pH 7.5) containing 100 mM KCl were mixed, heated and kept at 95 °C for 5 min, cooled back to room temperature before adding smart ligand (PyroTASQ) to the desired final concentration. Samples were then incubated at 4 °C for 12 h before electrophoresis. The gel was run in 90 mM TBE buffer with 100 mM KCl at 140 V for 20 min, dried and then imaged using a phosphorimager.

### Isolating and visualizing the PyroTASQ/c-di-GMP aggregates

All samples were prepared as follows: 50 mM Tris-HCl (pH 7.5), 100 mM KCl and c-di-GMP or GMP stock solutions were combined and mixed thoroughly in total volume of 100 μL. Solutions were heated to 95 °C on a heat block and kept at 95 °C for 5 min. The samples were then cooled down at room temperature for 15 minutes before the smart ligand (PyroTASQ) was added to a desired final concentration. Samples were then incubated at 4 °C for 12 h. After the incubation period, samples were centrifuged at 14,000 rpm for 5 min at 4 °C. The supernatant (100 μL) was carefully removed for fluorescence measurement. Fresh buffer (100 μL) was added to the decanted tube, heated to 95 °C to disaggregate the c-di-GMP/PyroTASQ precipitate and the solution containing the re-dissolved PyroTASQ fluorophore was transferred into a cuvette for fluorescence measurements. Each measurement was repeated three times.

## Computational part

All quantum level calculations were performed with the Amsterdam Density Functional (ADF)^49^,^50^ using dispersion-corrected density functional (BLYP-D3)^51^. Previous benchmark study on stacked DNA bases showed that it is essential to incorporate the dispersion correction^52^. For full system, where the pyro-core and the Gu-monomers are linked, the optimizations were performed with DZP basis set, while the analyses of the full and simplified systems as well as the optimization of the simplified systems were calculated with TZ2P basis set. The conformational space of the full molecule was sampled by a quick conformational search using the MacroModel program from the Schrodinger suit^53^. OPLS_2005 force field^54^ and implicit water model was applied; and the energy window of different structures was set to 5 kcal/mol. Conformers were always minimized at molecular mechanical level with 0.05 gradient convergent criteria.

## Additional Information

**How to cite this article**: Zhou, J. *et al*. Computational understanding and experimental characterization of twice-as-smart quadruplex ligands as chemical sensors of bacterial nucleotide second messengers. *Sci. Rep.* **6**, 33888; doi: 10.1038/srep33888 (2016).
